# Dietary Isoflavones and Breast Cancer Risk

**DOI:** 10.3390/medicines4020018

**Published:** 2017-04-07

**Authors:** Samira Ziaei, Reginald Halaby

**Affiliations:** Department of Biology, Montclair State University, 1 Normal Avenue, Montclair, NJ 07043, USA; ziaeis@montclair.edu

**Keywords:** diet, phytoestrogens, isoflavones, soy, breast cancer

## Abstract

Breast cancer is the deadliest neoplasm in women globally, resulting in a significant health burden. In many cases, breast cancer becomes resistant to chemotherapy, radiation, and hormonal therapies. It is believed that genetics is not the major cause of breast cancer. Other contributing risk factors include age at first childbirth, age at menarche, age at menopause, use of oral contraceptives, race and ethnicity, and diet. Diet has been shown to influence breast cancer incidence, recurrence, and prognosis. Soy isoflavones have long been a staple in Asian diets, and there appears to be an increase, albeit modest, compared to Asian populations, in soy consumption among Americans. Isoflavones are phytoestrogens that have antiestrogenic as well as estrogenic effects on breast cancer cells in culture, in animal models, and in clinical trials. This study will investigate anticancer and tumor promoting properties of dietary isoflavones and evaluate their effects on breast cancer development. Furthermore, this work seeks to elucidate the putative molecular pathways by which these phytochemicals modulate breast cancer risk by synergizing or antagonizing the estrogen receptor (ER) and in ER-independent signaling mechanisms.

## 1. Introduction

Despite recent medical advances, the mortality and incidence rates for cancer continue to be significant throughout the world. In particular, breast cancer is the deadliest neoplasm in women globally [1]. An estimated 14 million new cases and roughly 8 million deaths occur each year worldwide [2]. However, breast cancer is not limited to women only, it also affects men [3,4], transgender individuals [5,6], and people from all racial and ethnic backgrounds [7,8]. Clearly, there is a need for more novel, effective, and affordable treatments against breast cancer. Data from several studies provide support for a correlation between diet and cancer rates [9,10,11,12]. Furthermore, dietary factors may account for 30%–35% of all cancers [13]. Consumption of nutrients that contain elevated amounts of animal or caloric content, refined sugars, and alcohol increase cancer risk [14,15,16,17]. In contrast, phytochemicals are thought to exert anticancer effects through various molecular pathways, such as free radical scavenging, modulation of hormonal and enzymatic activities, and induction of DNA damage in cancer cells [18,19,20,21,22].

Phytoestrogens are naturally occurring plant compounds that are structurally similar to estrogens [23]. Five major classes of phytoestrogens have been described: flavonoids, isoflavonoids, lignans, coumestans, and stilbenes [23]. This review aims to clarify the relationship between soy isoflavones and breast cancer risk. Dietary isoflavones are present in various food items including beans, lentils, and soybeans [24]. The amount of dietary isoflavonoids consumed is geographically dependent. For instance, the mean daily isoflavone intake of 30 to 50 mg among older individuals in Japan [25], whereas in the United States [26], and Europe [27], per capita intake is less than 3 mg. The traditional Asian diet first-generation soy foods (e.g., tofu, tempeh, miso, and soymilk), while Western diets include other food sources that contain soy: soy-based meat derivatives and meat products with added soy protein [28]. Many of these second-generation soy items contain several-fold more isoflavone content than traditional Asian soy products [29]. Due to their chemical structure, the isoflavones can bind to estrogen receptors [30]. As a result of this binding, these phytoestrogens have been shown to inhibit [31] and promote [32] the expression of estrogen-sensitive genes.

Several reports indicate that the occurrence of breast cancer is considerably lower in Asian individuals compared with other populations because they incorporate high levels of isoflavones as part of their regular diet [33,34]. Verheus et al. found that high plasma levels of genistein were associated with reduced breast cancer risk in Dutch women [35]. There is a 9-fold difference between the daily amounts of soy isoflavones consumed by Chinese-Americans (4 g/day) compared to Chinese natives (36 g/day) [36]. The anticancer effects of dietary isoflavones in Asian women may also be attributed to early exposure to soy. Several reports demonstrate that high soy consumption during childhood may reduce one’s risk of developing breast cancer later in life and that the risk may be further reduced by soy intake as an adult [37,38,39,40].

The interest in phytoestrogens is also increasing for other reasons. They may provide a safer alternative to hormone replacement therapy in postmenopausal women [41]. Indeed, it has been reported that *S*-equol, a metabolite of daidzein, was shown to ameliorate menopausal symptoms [42]. In the United States, women have approximately a 14% chance of being afflicted by breast cancer during their lifetime [2], therefore the notion that by making a relatively low-cost lifestyle modification and ingesting an isoflavone-rich diet may reduce breast cancer is quite attractive. The use of soy isoflavones as potential treatments for cancer is also related to their antineoplastic properties. The isoflavones can block tumorigenesis by the following mechanisms: inhibiting enzymes required for DNA replication, metastasis, and signal transduction; disabling growth factors that promote angiogenesis, such as VEGF; and activating the immune system [30,31]. The precise mechanisms by which dietary isoflavones regulate breast cancer are not fully understood. In this review, we examine the protective as well as harmful effects that these phytoestrogens may have on breast cancer.

## 2. Isoflavones and Breast Cancer Prevention

Estrogen is known to induce breast cancer progression, and interventions, such as dietary modifications, that block or reduce estrogen production are likely to result in favorable prognoses for breast cancer patients. Yamamoto and colleagues demonstrated, in a population-based, prospective study, that frequent isoflavone consumption was inversely associated with the risk of breast cancer [43]. In another study, researchers found that soy isoflavones did not have estrogenic effects in humans and concluded that such a diet may be safe for breast cancer survivors [44]. These findings suggest that dietary soy may be chemoprotective and prevent recurrence in breast cancer patients.

It has been reported that isoflavones can suppress breast cancer cells in vitro and in vivo. Genistein inhibited invasion of breast carcinoma cells and inhibited tumor growth in nude mice bearing MCF-7 and MDA-MB-231 xenografts [45,46]. This inhibition was accompanied by downregulation of matrixmettaloproteinase-9 (MMP-9), a gene involved in tumor cell migration [46]. Daidzein’s anti-proliferative effects are associated with its ability to induce apoptosis, by increasing the Bax/Bcl-2 ratio, its antioxidant properties, and ability to inhibit cytokines and cyclin-dependent kinases (Cdk) [47,48]. Valachovicova et al. demonstrated that daidzein’s ability to inhibit the migration and invasion of MDA-MB-231 cells was mediated through NF-kappaB-dependent signaling pathways [49]. The isoflavones are able to inhibit a key enzyme, 17β-hydroxysteroid dehydrogenase-type 1, that is required to convert estrone to its active form, estradiol [50,51]. Additionally, isoflavones were found to inhibit angiogenesis by decreasing microvascular density, reducing circulating levels of VEGF, and increasing endostatin levels [52].

Genistein is a natural isoflavone phytoestrogen that has been observed to act as an estrogen mimetic based on structural similarities to the endocrine hormone [53]. The majority of breast cancer patients have ER–positive breast cancer [54]. This is another major reason why a clearer understanding of how isoflavones binding to ER may be used to develop novel treatment modalities. Genistein induces programmed cell death in cell lines with differential ER status, suggesting that it may be used as an adjuvant for breast cancer treatment.

**Figure 1:**
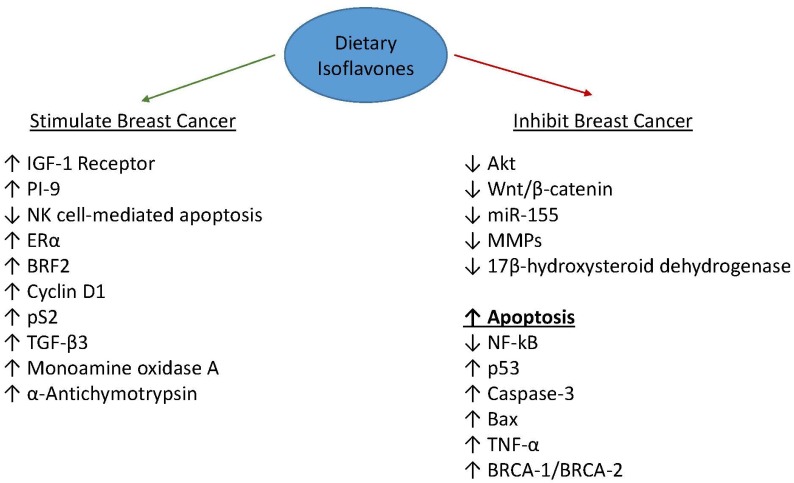
Proposed molecular mechanisms of stimulatory and inhibitory effects of dietary isoflavones on breast cancer.

For example, genistein induces apoptosis in breast carcinoma cell lines that are ER (+) and ER (−) [49,55,56]. Genistein’s ability to promote apoptosis requires wild-type caspase-3 activity [57]. This was demonstrated in experimental cells by introducing functional caspase-3 into the caspase-3 deficient cell line, MCF-7 and by mutating the protein in MDA-MB-231 cells [57]. Furthermore, a recent report demonstrated that genistein induced apoptosis in breast cancer cells by reducing expression levels of miR-155, an oncogenic micro-RNA that is expressed in breast tumors [58]. Taken together, these studies suggest that genistein’s growth inhibitory effects proceed via activation of apoptotic pathways. These data also demonstrate that genistein can execute its anticancer effects via estrogen-independent signaling pathways (Figure 1). Genistein, when administered in conjunction with adriamycin, was found to induce necrotic-like cell death in breast cancer cells by inactivating the HER-2 receptor [59]. Tamoxifen is usually the chemotherapeutic agent of choice for ER (+) breast cancer [60]. Unfortunately, not all ER (+) breast cancers respond to tamoxifen. In light of this, Mai and coworkers showed that genistein might be a viable treatment option for these tamoxifen-resistant breast tumors [61]. Genistein is thought to play a role in inhibiting the invasiveness and metastatic potential of MCF-7 and MDA-MB-231 cells presumably by downregulating the transcription of various matrix metalloproteinases (MMP) [62]. Likewise, many of the anticancer properties of genistein are believed to proceed through its regulation of various molecular signaling pathways that involve various genes, such as Bcl-2, Bax, NF-kB, and Akt [63]. BRCA-1 and BRCA-2 have been utilized as markers in various treatments for breast cancer [64,65]. Genistein can induce apoptosis in BRCA-1 wild type and BRCA mutant cancer cells [66]. Another study reported that genistein exposure results in the upregulation of BRCA-1 and BRCA-2 genes in a dose-and time-dependent manner [67]. Furthermore, genistein upregulates the expression of p53 and tumor-necrosis factor α genes in breast cancer cells [55,66,67]. A recent report showed that genistein-mediated downregulation of miR-155, a novel oncogenic microRNA, contributes to the anticancer effects of genistein in metastatic breast cancer [58]. Studies have investigated the effects of isoflavones on breast cancer risk when these compounds are administered at the start of puberty. Gallo and coworkers found that rats whose diet was supplemented with 0.7% soy demonstrated statistically significant higher rates of mammary gland tumors compared to control animals [68]. Likewise, Gotoh et al. found that animals fed a 10% miso diet, beginning at puberty, showed a significant reduction in the amounts of mammary tumors per rat [69]. Another study that examined soy consumption during puberty also detected a reduction in induced tumors in experimental animals who were fed a soy diet containing normal levels of isoflavones [70]. Shu et al. conducted a study that looked at consumption of soy during adolescence and breast cancer risk [37]. They found that consumption of soyfood between the ages of 13 and 15 years resulted in reduced risk of breast cancer later in life [37]. The data from a study by Wu et al. also support an inverse association between soy intake during adolescence and breast cancer risk [38].

## 3. Isoflavones and Breast Cancer Promotion

Because dietary soy phytoestrogens can also exert estrogenic effects, this has caused apprehension in clinicians and patients when these agents are being considered as possible therapeutic options. This concern is not unfounded, and there is evidence to support this notion. Allred and coworkers showed that various concentrations of genistein induced neoplastic growth in nude mice bearing MCF-7 xenograft tumors in a dose-dependent fashion [71]. Hsieh et al. reported that 100 nM of genistein stimulated proliferation of MCF-7 cells in vitro and in the mammary glands of mice bearing MCF-7 tumors [72]. These findings suggest that genistein’s estrogenic effects are not limited to cultured cells, rather they also affect normal breast tissue. It should be noted that when breast carcinoma cells were exposed to physiological concentrations of estrogen prior to implantation, rather than pharmacologic concentrations, isoflavones did not affect tumor growth [42]. In contrast, Ju et al. showed that dietary consumption of another isoflavone, daidzein, was able to stimulate growth of MCF-7 cells implanted in athymic mice [73]. Likewise, Johnson et al. also demonstrated that daidzin induced cell proliferation in breast cancer cells [74]. Daidzin’s tumor-promoting effects appear to proceed via the estrogen receptor. Specifically, Isoda et al. showed that daidzin binds to ER and that this binding was prevented by tamoxifen treatment [75].

Genistein induces proliferation of breast cancer cells in vitro. In one study, genistein specifically triggered the growth in ER (+) cells, T47D and MCF-7, but did not affect growth in ER (−) cells, MDA-MD-435 [76]. These results suggest that genistein’s tumorigenic effects are modulated via the ER. Another report found that dietary genistein was able to promote growth in mammary glands of ovariectomized athymic mice as well as in cultured MCF-7 cells [72]. A greater rate of mammary tumors was detected in erbB-2/neu mice fed a low-genistein diet and treated with tamoxifen compared with tamoxifen-treated mice fed a casein diet [77]. This finding was replicated in a study by Limer and colleagues [78]. Limer’s group showed that low concentrations of genistein stimulated growth in tamoxifen-sensitive cells, while growth was inhibited in tamoxifen-resistant cells [78]. These results suggest that low concentrations of dietary genistein may block the positive therapeutic effects of tamoxifen. If genistein concentrations, albeit low, are able to stimulate cell proliferation of breast carcinoma cells, one would presume that it could affect cell cycle-related genes. Dees et al. showed that dietary genistein promoted MCF-7 cell growth by increasing cyclin-dependent kinase 2 activity and cyclin D1 synthesis [79]. In summary, several studies demonstrate that physiological concentrations of dietary genistein induce growth of ER (+) breast cancer cells in both in vitro and in vivo models [53,80]. The concentration of genistein used in those studies mimicked human exposure to dietary isoflavones [37,38]. Taken together, the results lend support to the assertion that genistein’s estrogenic effects, such as proliferation of breast cancer cells and deregulation of the cell cycle, proceed through the estrogen receptor.

## 4. Genistein Acts via the Estrogen Receptor

Numerous lines of evidence indicate that the tumorigenic effects of genistein occur via interaction with the ERα [76,81,82]. Genistein presumably binds to ER to activate estrogen-dependent genes, such as TGF-β3, monoamine oxidase A (promotes metastasis), and α1-antichymotrypsin in vitro [83]. α1-antichymotrypsin is synthesized by MCF-7 cells in vitro and is postulated to play a role in the proteolytic degradation that accompanies metastasis [84]. In addition, genistein contains a ligand-binding domain that is similar in structure to that of 17-β estradiol [76]. Genistein is capable of abrogating the therapeutic effects of tamoxifen. As noted earlier, proliferation of mammary gland tumors were detected in mice fed a low-dose genistein diet and treated with tamoxifen [77]. Likewise, Limer and colleagues observed cell proliferation in tamoxifen-responsive cells (MCF-7 cells) treated with genistein [78]. Genistein’s modulation of the ER may explain how it exerts the following actions: proliferation of ER (+) cells, inhibition of tamoxifen’s effects, and activation of cell cycle genes [72,77,79,80]. Genistein-treated cells are able to evade apoptosis. Jiang et al. reported that genistein increased proteinase inhibitor 9 (PI-9) mRNA and protein levels and PI-9 inhibited apoptosis of MCF-7 cells by natural killer cells [85]. The epigenetic effects of isoflavones on ERα expression have been examined. Li et al. demonstrated that genistein treatment was able to upregulate ERα mRNA and protein expression in an ERα-negative cell line, MDA-MB-231 [86]. Similarly, Berner et al. found that genistein significantly hypermethylated the ERα promoter in colon cancer cells [87]. These findings suggest that restoration or maintenance of functional ERα may serve as a key target for genistein-related anticancer treatment options.

## 5. Human Studies

The signaling pathways that modulate the beneficial effects of dietary soy isoflavones on breast cancer have yet to be fully elucidated. The findings from several reports indicate that soy isoflavones can improve prognosis in breast cancer patients. One large cohort study of over 11,000 patients revealed that soy food consumption may be a potential treatment option, especially for ER (−) breast cancer in postmenopausal women [88]. Zhang et al. showed that genistein intake mimicking Asian consumption patterns improved the response of mammary tumors to tamoxifen therapy, and this effect was linked to reduced activity of unfolded protein response (UPR) and prosurvival autophagy genes and increased antitumor immunity [89].

In human studies, the implementation of dietary isoflavones for breast cancer patients has been somewhat controversial, in light of their dual nature (i.e., estrogenic and antiestrogenic actions). However, Chi et al. conducted a large, meta-analysis study and found that soy intake was correlated with reduced incidence and mortality of breast cancer [88]. Likewise, Guha and colleagues observed reduced breast cancer recurrence with increasing amounts of daidzein consumption in a prospective cohort study of postmenopausal women who were treated at some point with tamoxifen [90]. A significant number of breast cancer cases arise in postmenopausal women [91]. Chemotherapeutics are known to induce side effects that are similar to the symptoms of menopause in breast cancer patients [92]. Lu et al. observed that women who consumed dietary soy consistently for 1 full month had decreased plasma levels of estradiol and detected a 3-day average increase in the length of their menstrual cycles [93]. The reduced circulating estradiol levels may be responsible, in part, for genistein’s ability to reduce breast cancer risk [93]. A prospective study conducted by Shu et al. showed that women who continued to consume soy after being diagnosed with breast cancer had significantly lower levels of recurrence compared with women who consumed little to no soy [94]. We do not fully comprehend how the consumption of soy isoflavones precisely affects breast cancer recurrence. However, we can speculate that the outcome is dependent on differential gene expression induced by the isoflavones. For example, Maskarinec et al. found that women who consumed high amounts of dietary soy in early life had lower levels of HER2/neu and PCNA staining in malignant breast tissue [95].

## 6. Conclusions

We conclude that dietary isoflavonoids possess both antiestrogenic and estrogenic effects on breast cancer cells. Several plausible explanations can be offered. Opposing effects (cell proliferation and apoptosis) of isoflavones on breast cancer cells have been detected in vitro and in vivo. This might be due to the fact that cells exposed to isoflavones in culture respond quite differently than their cellular counterparts in animal models. Furthermore, the signaling pathways that are available to cultured cells are presumably different than those present in a whole animal. Another contributing factor is the lack of a universal or standard isoflavone diet. Rather, there is quite a broad range of food sources that contain isoflavones. This fact makes it difficult when comparing and analyzing data from studies that use various concentrations and different soy isoflavone constituents. It is evident that differences in the source and amount of soy isoflavones consumed by Chinese versus Chinese-American or American women can greatly impact breast cancer. The phytochemicals present in different isoflavones (e.g., genistein and daidzein) can amplify or dampen signaling pathways leading to opposing outcomes on breast cancer risk.

Breast cancer continues to be a global affliction, killing men, women, transgender individuals, teens through the elderly, and individuals from every racial and ethnic background. Novel therapeutic approaches, including dietary changes, need to be explored. Further studies, especially large clinical trials, are warranted to better inform patients, clinicians, and scientists concerning the safety of using dietary soy isoflavones as stand-alone or adjuvant treatments for breast cancer. These clinical trials should attempt to minimize or eliminate biases in order to ensure the accuracy and reproducibility of these findings. For instance, special attention must be given to the total diet of the participants. It has been shown that consumption of berries and peaches is correlated with a reduced risk of ER-negative breast cancer [96]. Additionally, there are some non-soy food items that contain isoflavones, such as asparagus and pinto beans [97]. Researchers need better guidelines to decide whether non-soy but isoflavone-containing foods will be included in their study protocols. There are also discrepancies between the type of soy food products that have been examined in the epidemiological studies. In particular, some studies chose soyfood [37,43,98], while others chose soy protein [99,100,101]. Clearly, the aforementioned criteria can affect the results of clinical studies. Finally, the developmental period when consumption of isoflavones starts affects breast cancer risk. As was discussed earlier in this review, dietary soy intake during early life provides protection against breast cancer [37,38]. This notion is also supported by animal studies in which animals ingested soyfoods prepubertally or perinatally [102,103]. This may explain why women who start eating soy products as adults, as is often the case in the Western world, lack the chemoprotective effects observed in Asian women.
